# The importance of non-pharmacological approach versus pharmacological treatment of behavioral and psychological symptoms (bpsd) in patients with Alzheimer’s dementia (AD) in a geriatric institution

**DOI:** 10.1192/j.eurpsy.2024.1316

**Published:** 2024-08-27

**Authors:** M. Spirova

**Affiliations:** ^1^PHI Specialized Hospital for Geriatric and Palliative Medicine, Skopje; ^2^PHI General hospital, Kavadarci; ^3^PHI Health center Skopje, Skopje, North Macedonia

## Abstract

**Introduction:**

BPSDs in patients with AD are present up to 90% and can cause serious complications in their overall health. A non-pharmacological approach and cognitive enhancers should be a priority in treatment in order to reduce the use of antipsychotics. In the pharmacological treatment of bpsd, additional therapy is inevitable in many cases

**Objectives:**

the need for adequat educatation of the medical staff in a geriatric center for the nonfarmacological approach in patients with bpsd in AD. Polypharmacy is common in farmacological treatment.

**Methods:**

A cross-sectional study of 180 patients hospitalized at geriatric unit in period of January till May 2023 was conducted. 61(33.9%) were patients with AD, 44 or 72.1% were females and 17 or 27.9% were males, with mean age 78.6±5.6 years. 50 patients (82.0%) had potentiated BPSD in the first days of hospitalization and needed additional therapy

**Results:**

: 19 od 61pts (31.1%) were on dual therapy, full doses of donepezil and memantine. 17 (89.5%) needed additional therapy for BPSD; 13 (68.4%) a short-term antipsychotic and 4 (21.1%) patients antidepressant therapy. 22 patients (36.1%) were admitted with donepezil only. 18 (81,8%) needed additional therapy. The remaining 20 (32,8%) were solely on memantine. 15 (75.0%) needed additional therapy

**Conclusions:**

Vast majority of patients AD (82.0%) manifested BPSD and needed additional therapy. Number of scientific papers it is found that cognitive stimulation in persons with moderate dementia has a benefit more than any pharmacological treatment. Education of caregivers of people with AD is inevitable.Opening of day care centers that will enable continuous support as well as individual access which would help delay institutionalization of people with BPSD at ADthe need

**Disclosure of Interest:**

None Declared

